# Characterization and protective activity of monoclonal antibodies directed against Fe (3+) ABC transporter substrate-binding protein of *Glaesserella parasuis*

**DOI:** 10.1186/s13567-021-00967-1

**Published:** 2021-07-05

**Authors:** Kexin Zhu, Dong Yu, Jiahui An, Yufeng Li

**Affiliations:** Key Laboratory of Bacteriology, Ministry of Agriculture, College of Veterinary Medicine, Nanjing Agricultural University, Nanjing, 210095 China

**Keywords:** *Glaesserella parasuis*, Monoclonal antibody, Complement-killing effect, Opsonization, Subunit vaccine candidate

## Abstract

Glässer’s disease is caused by the agent *Glaesserella parasuis* and is difficult to prevent and control. Candidate screening for subunit vaccines contributes to the prevention of this disease. Therefore, in this study, the inactivated *G. parasuis* reference serovar 5 strain (*G. parasuis*-5) was used to generate specific monoclonal antibodies (mAbs) to screen subunit vaccine candidates. Six mAbs (1A12, 3E3, 4C6, 2D1, 3E6, and 4B2) were screened, and they all reacted with the *G. parasuis* serovar 5 strain according to laser confocal microscopy and flow cytometry (FCM). Indirect enzyme-linked immunosorbent assay (ELISA) showed that one mAb 2D1, can react with all 15 reference serovars of *G. parasuis*. Protein mass spectrometry and Western blot analysis demonstrated that mAb 2D1 specifically reacts with Fe (3+) ABC transporter substrate-binding protein. A complement killing assay found that the colony numbers of bacteria were significantly reduced in the *G. parasuis*-5 group incubated with mAb 2D1 (*p* < 0.01) in comparison with the control group. Opsonophagocytic assays demonstrated that mAb 2D1 significantly enhanced the phagocytosis of 3D4/21 cells by *G. parasuis* (*p* < 0.05). RAW264.7 cells with stronger phagocytic ability were also used for the opsonophagocytic assay, and the difference was highly significant (*p* < 0.01). Passive immunization of mice revealed that mAb 2D1 can eliminate the bacteria in the blood and provide protection against *G. parasuis*-5. Our study found one mAb that can be used to prevent and control *G. parasuis* infection in vivo and in vitro, which may suggest that Fe (3+) ABC transporter substrate-binding protein is an immunodominant antigen and a promising candidate for subunit vaccine development.

**Supplementary Information:**

The online version contains supplementary material available at 10.1186/s13567-021-00967-1.

## Introduction

*Glaesserella parasuis* (*G. parasuis*) is a resident of the upper respiratory tract of normal pigs and the causative agent of Glässer’s disease. This disease is characterized by fibrinous polyserositis, arthritis, meningitis, and frequent symptoms of pneumonia, which often occur in pigs under stress or infected with other pathogens [1]. Although 15 serovars have been identified, 26.2% of the isolated strains cannot be typed [2]. Serovar 5 is thought to be highly virulent, and epidemiological studies have shown that it is the most prevalent in China [2, 3].

To date, the prevention and control of Glässer’s disease have mainly focused on the application of antibiotics and vaccines. Misuse of antibiotics has led to antimicrobial resistance (AMR), and the rate of AMR in bacteria has increased from 2008 to 2017 in China [4, 5]. AMR is also a serious threat to animal and public health, and international policies are demanding a reduction in their use, especially in the farming industry [6]. In this context, vaccination has become a better plan to prevent Glässer’s disease. However, there is a problem with the existing vaccines, which can provide good homologous protection but heterologous protection has been variable [7].

Screening of immunodominant antigens is key to designing a targeted immune response against virulent *G. parasuis* [8]. Tadjine [9] used the *G. parasuis* serovar 4 strain as an immunogen to prepare two mAbs. Among them, mAb 4D5 recognizes the epitope of the outer membrane protein, and mAb 4G9 recognizes the epitope of the lipopolysaccharide. Through passive immunization protection experiments, they found that the two mAbs can provide immune protection in mice. Tian et al. [10] also used inactivated whole bacteria of the local isolate HLJ-018 to immunize BALB/c mice, and mAb 1D8 against the outer membrane protein OmpA was obtained. mAb 1D8 with opsonization can react with 1–15 serovars of *G. parasuis* and protect mice from homologous and heterologous *G. parasuis* strains. Many studies have shown that lipopolysaccharides and outer membrane proteins are virulence-related factors of bacteria and have strong immunogenicity [11].

In this study, the *G. parasuis* reference serovar 5 strain (*G. parasuis*-5) was inactivated to immunize BALB/c mice to prepare six mAbs. By mass spectrometry analysis, three proteins were identified and expressed, and the specific antigen targeted by mAb 2D1 was confirmed as Fe(3+) ABC transporter substrate-binding protein through Western blot analysis. The characterization and protective activity of mAb 2D1 was confirmed in vitro and in vivo. Using mAb 2D1, we first indicate that Fe(3+) ABC transporter substrate-binding protein could be a promising novel candidate for subunit vaccine development, which will lay a theoretical foundation for the immunological prevention and treatment of Glässer’s disease.

## Materials and methods

### Reference strains, cells, animals and main reagents

Fifteen reference serovars of *G. parasuis* (Additional file 1), SP2/0 cells, and 3D4/21 cells were kept by our laboratory. Polyclonal antibodies against *G. parasuis*-5 (positive serum prepared by immunizing rabbits) and mAb against *Actinobacillus pleuropneumoniae* ApxIV (mAb ApxIV) were preserved in our laboratory. Fetal bovine serum (FBS) was purchased from Science Cell. HAT (Hypoxantin, Aminopterin, Thymidin), HT (Hypoxantin, Thymidin), PEG4000, and Freund’s complete and incomplete adjuvants were all purchased from Sigma Biological Company. Eight-week-old female BALB/c and ICR mice were purchased from Shanghai Xipuer-Bikai Experimental Animal Co., Ltd. All animal assays were performed in the Laboratory Animal Center of Nanjing Agricultural University and were approved by the Department of Science and Technology of Jiangsu Province [permit number: SCXK (SU) 2012-0004].

### Preparation and subtype identification of mAbs

Eight-week-old female BALB/c mice were immunized with *G. parasuis*-5 (1 × 10^9^ CFU, inactivated with 0.3% formaldehyde at 37 °C for 24 h) three times at 2-week intervals. The immunization route consisted of multiple subcutaneous injections on the back. An intraperitoneal booster with the same dose of antigen was administered 3 days before fusion. The antibody levels of mice were examined by indirect ELISA. The mouse with the highest antibody titer was selected for cell fusion according to conventional methods [12]. The culture supernatants were also identified by indirect ELISA. The positive hybridomas were subcloned three or more times by limiting dilution. Ascites fluid was produced by intraperitoneal injection of positive hybridoma cells in liquid paraffin-treated BALB/c mice (8-week-old female BALB/c mice were inoculated intraperitoneally with 500 μL of sterile liquid paraffin). Subtype classes of mAbs were identified using a Mouse Monoclonal Antibody Isotype ELISA Kit (Proteintech, USA) according to the manufacturer’s protocol.

### Indirect ELISA

In brief, the microtiter plate (96-well, Corning, USA) was coated with 100 µL of inactivated *G. parasuis*-5 (2.24 × 10^8^ CFU/mL) at 37 °C for 2 h and then placed overnight at 4 °C. The next day, the plates were washed 3 times with phosphate buffer containing 0.05% Tween 20 (PBST) and placed at 37 °C with 5% skim milk (PBST as diluent) for 2 h. Next, the plates were washed three times. Supernatant of hybridioma culture was added to the wells and kept at 37 °C for 1 h (100 μL/well). At the same time, the sera from immunized and nonimmunized mice served as either positive or negative controls. After washing, 100 μL of goat anti-mouse IgG-HRP (H+L) antibody (diluted at 1:250, Shanghai Biyuntian Biotechnology Co., Ltd) was added and incubated at 37 °C for 45 min. The color was developed by the addition of 100 μL of TMB for 10 min and stopped by the addition of 2 M H_2_SO_4_. Finally, an optical density of 450 nm (OD_450_) was recorded using an ELISA plate reader (BioTek, USA). When the ratio of the positive value (*P*) to the negative value (N) is greater than 2.1 (P/N > 2.1), it can be judged as a positive sample.

Indirect ELISA was also carried out to detect the reactivity of six mAbs with 1–15 reference strains of *G. parasuis*. Microtiter plates were coated with 100 µL of inactivated 1–15 reference strains of *G. parasuis* as antigens. Six mAbs and positive or negative controls were used as primary antibodies, and goat anti-mouse IgM/HRP antibody (Beijing Boaosen Biotechnology Co., Ltd) was used as the secondary antibody. Other operations were as described above.

### Confocal laser assay

Sterile coverslips were placed into the bottom of 24-well plates and then inoculated with 3D4/21 cells (60% cell well confluency). Each cell well was inoculated with *G. parasuis*-5 at a dose of 400 MOI, and the cell plate was centrifuged at 800 × *g* for 10 min and then incubated at 37 °C for 1 h [13]. After washing 3 times with PBS, the infected cells on the coverslip were fixed with 4% formaldehyde. Six mAbs were used as the primary antibodies, and goat anti-mouse IgM/FITC antibody (Beijing Boaosen Biotechnology Co., Ltd) was used as the secondary antibody. After washing with PBS for 3 times, the coverslips were placed upside down on clean glass slides dripped with 10 μL of 50% glycerol, and they were mounted and observed under a laser confocal microscope (LSM780, Zeiss, USA).

### Flow cytometry (FCM)

Bacteria were labeled with a CFDA-SE cell proliferation and tracer detection kit (Shanghai Biyuntian Biotechnology Co., Ltd). The labeled bacterial solution was observed under a fluorescence microscope to check whether the bacteria were labeled with fluorescence. After confirmation, FSC and SSC were used to set up gates to delineate the area of the target bacteria in the scatter diagram. *G. parasuis*-5 cultured to the logarithmic phase was washed 3 times with PBS and then resuspended and fixed with formaldehyde for 20 min at 25 °C. The bacterial pellet was blocked with PBS containing 5% (v/v) skimmed milk for 0.5 h. Then, mAbs were added to bacterial pellets and incubated at 37 °C for 0.5 h. Goat anti-mouse IgM/FITC was added and incubated at 37 °C for another 1 h. Subsequently, the bacterial pellet was washed 3 times and resuspended in 300 μL of PBS for FCM analysis (CYTO FLUX, Beckman, USA).

### Identification of the target protein of mAb 2D1

Western blot analysis was used to identify proteins that specifically reacted with mAb 2D1. Whole bacterial proteins were separated by 12.5% SDS-PAGE and transferred to polyvinylidene difluoride membranes (Millipore). The membranes were blocked with PBST containing 5% skimmed milk for 2 h. mAb 2D1 was used as the primary antibody, and goat anti-mouse IgM/HPR was used as the secondary antibody. After four washes with PBST, the membranes were treated with Pierce ECL Western blotting Substrate (Thermo Fisher Scientific) and observed with the Tanon Chemiluminescent Imaging System (Biotanon, China).

Protein mass spectrometry was used to analyze the suspected target protein against mAb 2D1. After separating the bacterial proteins with 12.5% SDS-PAGE, the target protein band in the gel was cut out carefully and then sent to Gene Denovo Biotechnology Co., LTD., Guangzhou, for full spectrum analysis. According to the results of mass spectrometry, three proteins were selected for *E. coli* expression. Western blot experiments (the steps were the same as before) were carried out to identify the target protein of mAb 2D1.

### Expression of recombinant proteins

To obtain GAPDH, Fe(3+) ABC transporter substrate-binding protein and porin fusion proteins, three pairs of primers (Table 1) were used to amplify the full-length sequences of GAPDH, Fe(3+) ABC transporter substrate-binding protein and porin genes from *G. parasuis*-5. The amplified fragments of GAPDH and Fe(3+) ABC transporter substrate-binding protein were cloned into the pET-28a(+) expression vector, and the amplified porin fragment was cloned into the pET-32a(+) expression vector. The cycling conditions were 95 °C for 10 min, followed by 35 cycles of 95 °C for 30 s, 50 °C for 30 s, and 72 °C for 70 s. Finally, the cycling conditions were extended for 10 min at 72 °C. The PCR products were purified from agarose gel and cloned into pET-32a(+) or pET-28(+). The recombinant plasmids named pET-28a-GAPDH, pET-32a-porin and pET-28a-Fe(3+) ABC transporter substrate-binding protein were sequenced (Genscript, Nanjing, China). The positive recombinant plasmid was transformed into Rosetta and induced with 1 mM isopropyl β-d-1-thiogalactopyranopyranoside (IPTG) at 37 °C for 4 h. Bacterial cells were collected by centrifugation at 8000 × *g* for 10 min. Thereafter, GAPDH, Fe(3+) ABC transporter substrate-binding protein and porin fusion proteins were analyzed by sodium dodecyl sulfate polyacrylamide gel electrophoresis (SDS-PAGE).

**Table 1:** **Primers for amplification of the full-length GAPDH, porin and Fe(3+) ABC transporter substrate-binding protein genes**

Gene	Primer sequences (5′–3′)	Restriction sites	Length (bp)
GAPDH	GCGGATCCATGGCAATTAAAATTG	BamHI	1021
	GCAAGCTTTTAGCCTTTGTAGTTG	HindIII	
Porin	CGCGAGCTCAAAAAAACACTAGTAGCATTAGC	SacI	1081
	CGCGAGCTCAAAAAATCTCTTTCTGTGCTTGC	XhoI	
Fe (3+) ABC transporter substrate-binding protein	CGCGAGCTCAAAAAATCTCTTTCTGTGCTTGC	SacI	1020
	CGCCTCGAGTTTCGCACCGAAATCATCAAATT	SacI	

### In vitro complement killing assay and opsonophagocytic assay (OPA)

The complement killing assay was modified from previously published protocols [10]. *G. parasuis*-5 cultured to the logarithmic phase was diluted to 10^3^ CFU/mL and incubated with mAb 2D1 (complement-inactivated at 56 °C for 0.5 h) for 0.5 h at 37 °C. Positive serum against *G. parasuis*-5, PBS and mAb ApxIV were used as positive, mock and negative controls, respectively. Then, guinea pig serum (5%, v/v, undetected antibodies against *G. parasuis*, Shanghai Yuanye Biotechnology Co., Ltd) was added to the above four mixtures as a source of complement. After incubating at 37 °C for 2 h, the mixtures were evenly spread on TSA agarose plates and incubated at 37 °C for 48 h. Then, the colony numbers were counted. All assays were performed in triplicate and repeated 3 times.

To assess the opsonophagocytic activity of monoclonal antibodies, we performed experiments with slight modifications based on previous reports [14, 15]. Briefly, 3D4/21 cells were seeded into a 12-well plate (approximately 4 × 10^5^ cells/well). *G. parasuis*-5 (10^9^ CFU/mL) cultured to logarithmic phase was mixed with complement-inactivated mAb 2D1. After incubating at 37 °C for 0.5 h, the mixtures were added onto cells at 200 MOI. Then, 5% (v/v) guinea pig serum was added to each cell well and incubated at 37 °C in a 5% CO_2_ atmosphere for 2 h. Nonspecifically attached bacteria were removed by washing with PBS, and complete growth medium (including 100 U/mL penicillin G and 0.25 mg/mL gentamicin) was added to each cell well. The cell plate was incubated for another 1 h to kill extracellular bacteria. Subsequently, we used double-distilled water to lyse the cells, and lysates were evenly streaked onto TSA agarose plates. At the same time, positive serum, PBS and the mAb ApxIV were used as positive, mock and negative controls, respectively. All assays were performed in triplicate and repeated three times. In addition, we also used RAW264.7 cells to perform the same experiment, and the operation steps were as described above.

### Passive immunization with mAb in mice

Fifteen 6-week-old female ICR mice were randomly divided into 3 groups, with 5 in each group. One hour before the challenge, mAb 2D1, positive serum and mAb ApxIV were injected via the intraperitoneal pathway. Then, each mouse was intraperitoneally injected with 0.2 mL of *G. parasuis*-5 suspension (8.42 × 10^9^ CFU). Mice were observed for 7 days after the challenge. At 12 h, 24 h, 48 h, and 72 h, blood samples were collected from the tail vein of the surviving mice (with sterile operation). After being diluted with sterile PBS, the blood was applied to TSA solid medium and incubated at 37 °C for 24 h. At the same time, the survival rate of mice in each group was compared.

### Statistical analysis

GRAPHPAD version 5.01 was used for statistical analysis.

## Results

### Preparation and subtype identification of mAbs

After 3 screening and subcloning cycles, six hybridoma cell lines were obtained for expansion culture. All of them can secrete stable antibodies against *G. parasuis*-5. The subtypes of the six mAbs were all IgM/κ chains identified by the mouse monoclonal antibody subtype identification kit.

### Reactivity of mAbs to *G. parasuis*-5

**Figure 1:**
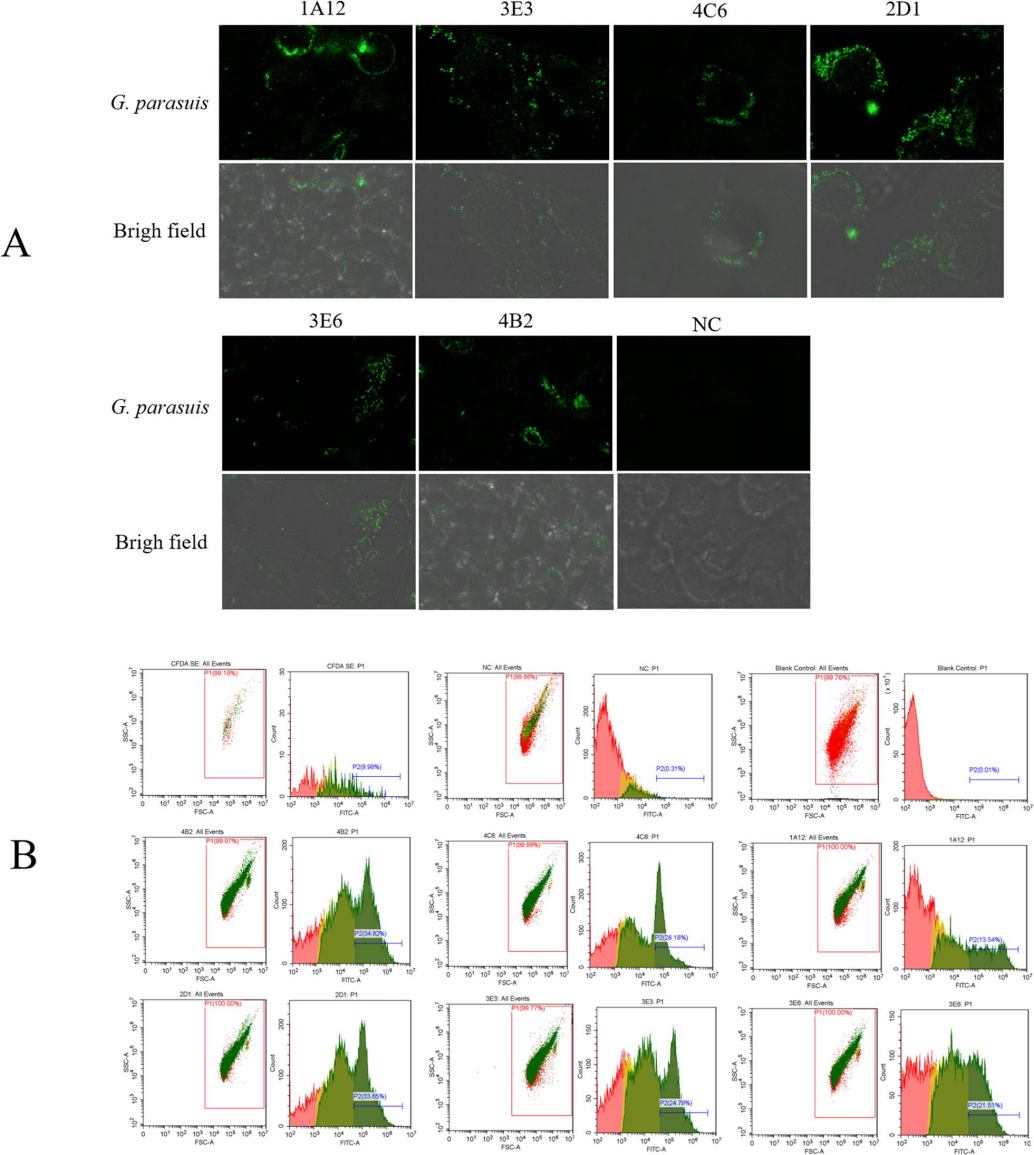
**Specificity of mAbs by confocal laser scanning microscopy and flow cytometry (FCM).** **A** 3D4/21 cells were infected with *G. parasuis*-5, six mAbs were added to the cell wells as primary antibodies and incubated at 37 °C for 1 h, and goat anti-mouse IgM/FITC antibody was used as the secondary antibody. After washing with PBS 3 times, the coverslips were placed upside down on clean glass slides dripped with 10 μL of 50% glycerol and mounted and observed under a laser confocal microscope. Fluorescence was observed in the cell wells with *G. parasuis*-5 and mAbs 1A12, 3E3, 4C6, 2D1, 3E6, and 4B2, and there was no fluorescence in the cell wells with *G. parasuis*-5 and the negative control. Scale bar = 5 μm. **B** *G. parasuis*-5 cells were labeled with a CFDA-SE cell proliferation and tracer detection kit. FSC and SSC were used to set up the gate to delineate the area of *G. parasuis*-5 in the scatter diagram. Six mAbs were used as primary antibodies, and goat anti-mouse IgM/FITC was used as the secondary antibody. They were all incubated with *G. parasuis*-5 and then used for FCM analysis. Fluorescence was observed in *G. parasuis*-5 incubated with six mAbs, but there was no fluorescence in *G. parasuis*-5 incubated with the negative control.

The confocal laser results showed that there was a clear fluorescence reaction in the cell wells after the mAb and *G. parasuis*-5 were incubated together, and no specific fluorescence was found in the negative control group (Figure 1A). This indicates that the six mAbs can all react with *G. parasuis-*5. The FCM results also verified this conclusion (Figure 1B).

### Reactivity of mAb 2D1 to *G. parasuis* reference serovars 1–15

**Figure 2:**
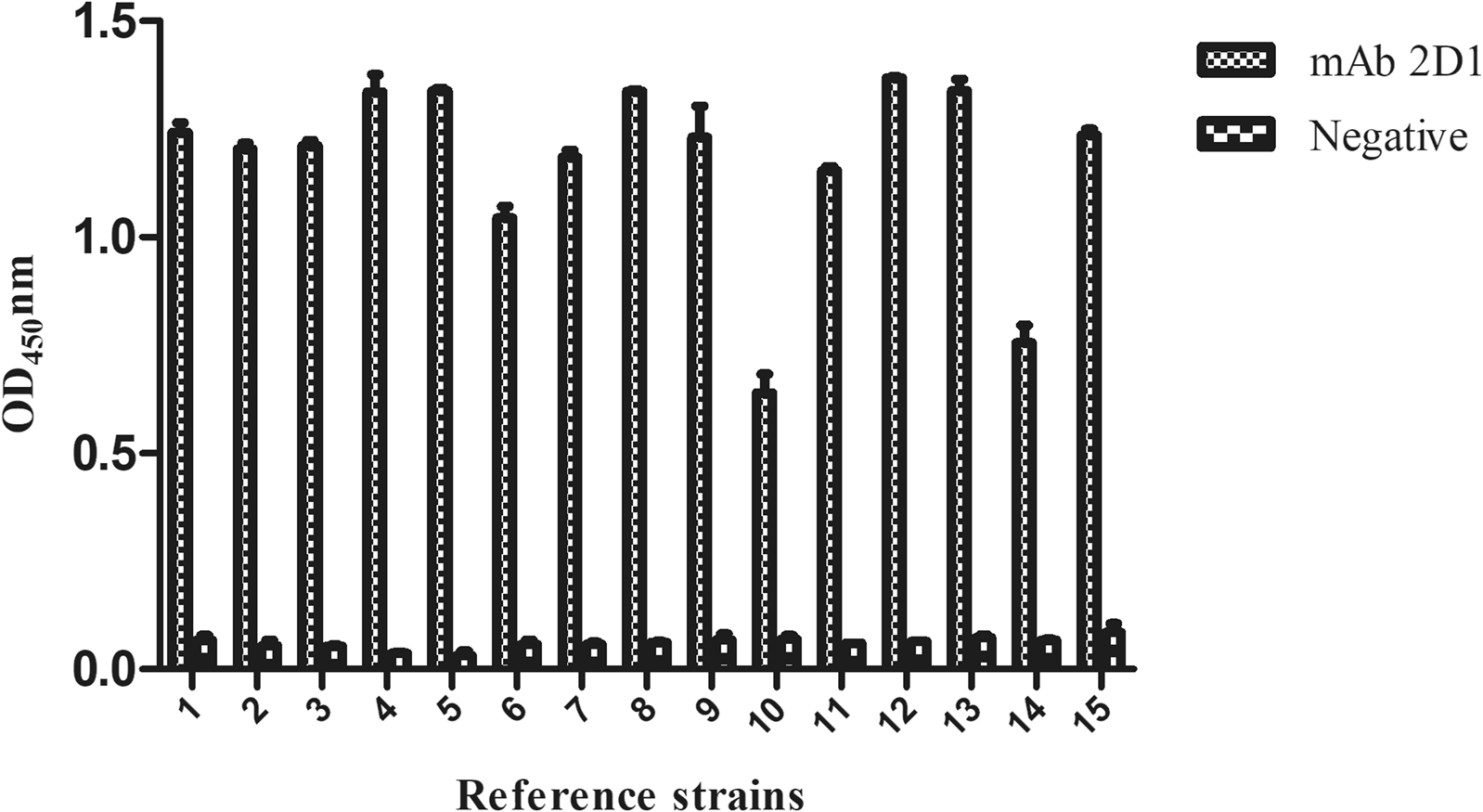
**Broad reactivity of mAb 2D1 with *G. parasuis* 1–15 reference serovars using indirect ELISA.** Reference serovars 1–15 of *G. parasuis* were used as the coating antigens, six mAbs were used as the primary antibody, and goat anti-mouse IgM/HRP antibody was used as the secondary antibody. The results of indirect ELISA showed that six mAbs can positively react with reference serovars 1–15 of *G. parasuis* and mAb 2D1 had the strongest reaction with bacteria (the figure only shows indirect ELISA results of mAb 2D1 and the results of other five mAbs tested with fifteen reference strains were not shown).

The results of indirect ELISA demonstrated that six mAbs can positively react with reference serovars 1–15 of *G. parasuis* (Additional file 2). In particular, mAb 2D1 had the strongest reaction with bacteria (Figure 2), and thus, mAb 2D1 was chosen for subsequent experiments.

### Expression and identification of targeted protein

**Figure 3:**
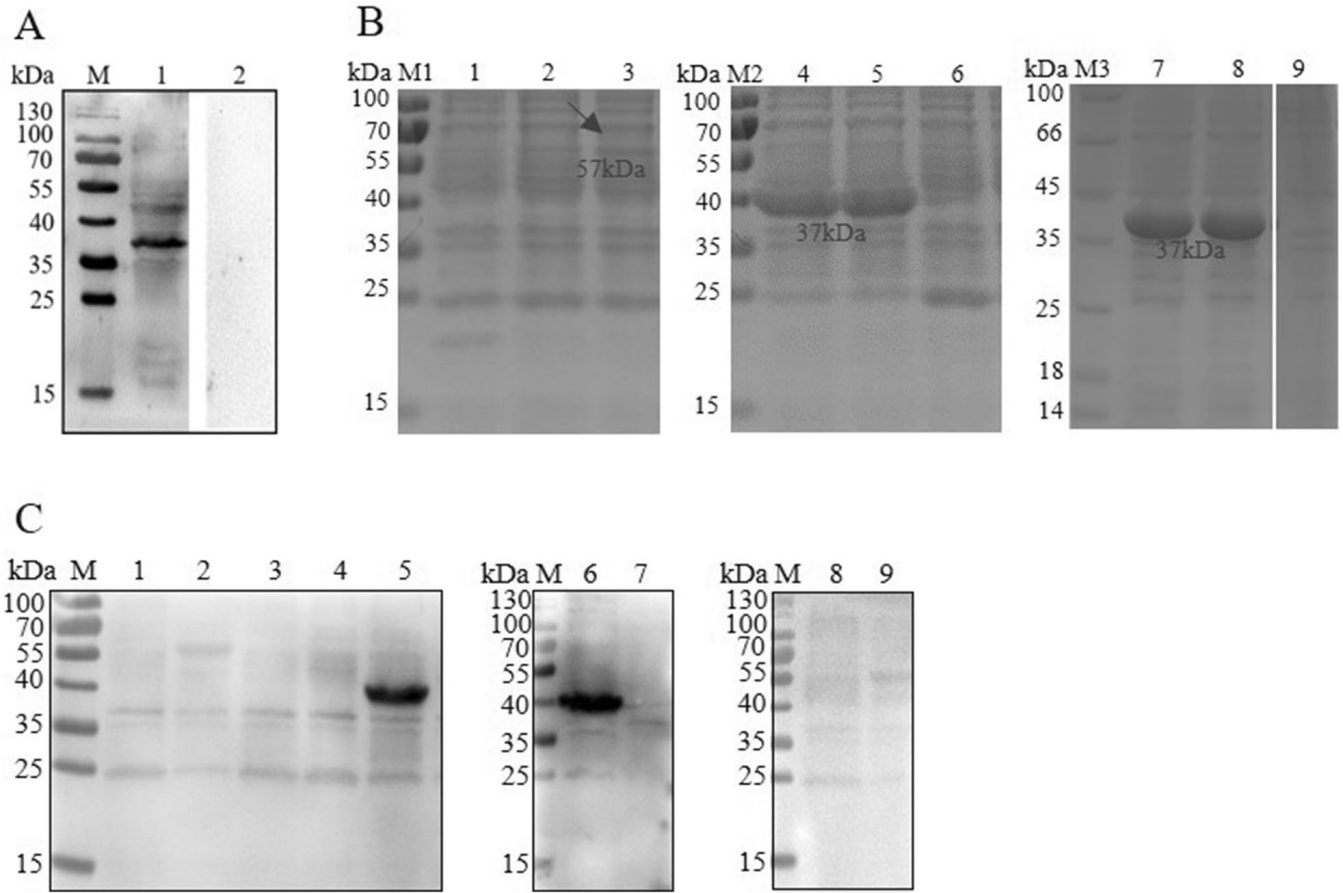
**Immunogens analysis of mAb 2D1.** **A** Reaction of *G. parasuis* with mAb 2D1 was identified by Western blot. M: Protein markers; lanes 1 and 2: *G. parasuis* serovar 5 strain (the primary antibodies for lane 1 were mAb 2D1 and for lane 2 were mAb ApxIV). **B** The expression of recombinant proteins was analyzed by SDS-PAGE. M1, M2, M3: Protein markers, lane 1: Rosetta containing pET-32a induced by IPTG; lanes 2 and 3: Rosetta containing pET-32a-porin induced by IPTG (the arrow indicates the position of the protein); lanes 4 and 5: Rosetta containing pET-28a-Fe(3+) ABC-transporter substrate-binding protein induced by IPTG; lane 6: Rosetta containing pET-28a induced by IPTG; lanes 7 and 8: Rosetta containing pET-28a-GAPDH induced by IPTG; lane 9: Rosetta containing pET-28a induced by IPTG (all were done with mAb 2D1 as the primary antibody). **C** The reactions of recombinant proteins with mAb 2D1 were analyzed by Western blot. Lane 1: Rosetta containing pET-32a induced by IPTG; lane 2: Rosetta containing pET-32a-porin induced by IPTG; lane 3: Rosetta containing pET-28a-GAPDH induced by IPTG; lane 4, 7: Rosetta containing pET-28a induced by IPTG; lanes 5 and 6: Rosetta containing pET-28a-Fe(3+) ABC-transporter substrate-binding protein induced by IPTG (all were done with mAb 2D1 as the primary antibody); lane 8: Rosetta containing pET-28a-Fe(3+) ABC-transporter substrate-binding protein induced by IPTG; lane 9: Rosetta containing pET-28a induced by IPTG (the primary antibody of lane 8 and lane 9 is mAb ApxIV).

Western blot analysis showed that the molecular weight of the protein reacting with mAb 2D1 was approximately 35–40 kDa (Figure 3A). Mass spectrometry showed that GAPDH, Fe(3+) ABC transporter substrate-binding protein and porin may be the target proteins of mAb 2D1 (Table 2). The SDS-PAGE results showed that all these proteins could be stably expressed (Figure 3B). Then, Western blotting was performed to identify proteins targeted by mAb 2D1. The Western blot analysis results showed that mAb 2D1 specifically reacted with Fe(3+) ABC transporter substrate-binding protein (Figure 3C), which indicates that this protein is an immunogen of mAb 2D1.

**Table 2:** **The results of mass spectrometry analysis**

Protein	Peptides	Unique	Coverage (%)	Gene	Gene ID
GAPDH	58	123	75	gap	7278596
Fe(3+) ABC transporter substrate-binding protein	123	154	95	HAPS_RS05495	7278853
Porin	154	58	80	HAPS_RS00830	7278006

### Complement activation effect of mAb 2D1 in vitro

**Figure 4:**
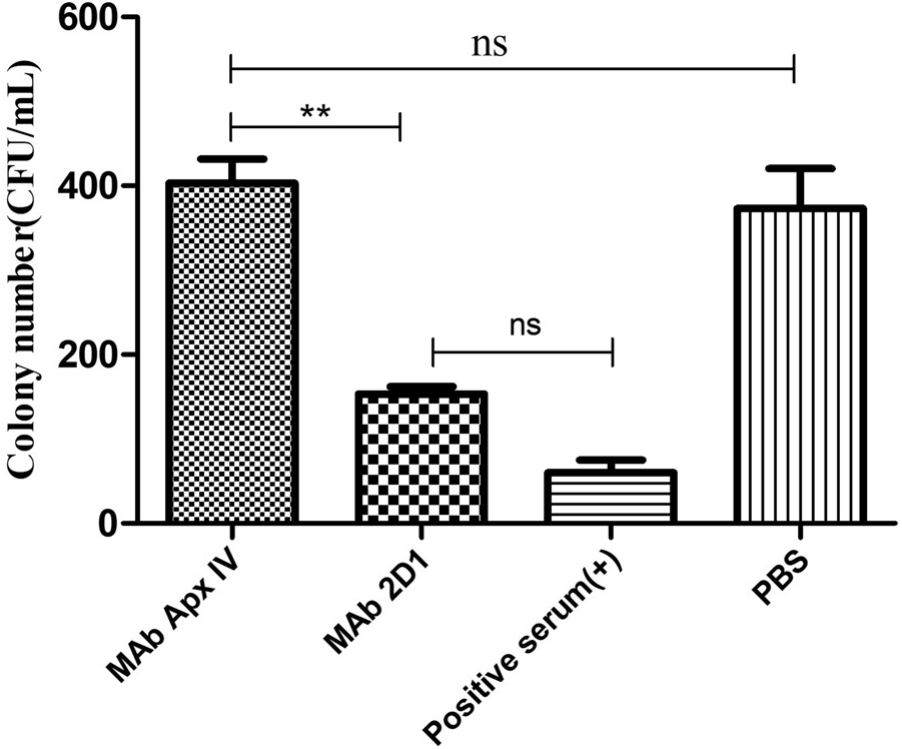
**Complement killing activity of mAb 2D1 in vitro.** The *G. parasuis* serovar 5 strain (diluted to 10^3^ CFU/mL with PBS) was incubated with mAb 2D1 (complement-inactivated), positive serum against *G. parasuis*-5 (prepared by immunizing rabbits and preserved in our laboratory), PBS and mAb ApxIV (mAb against *Actinobacillus pleuropneumoniae* preserved in our laboratory) at 37 °C for 2 h. The mixtures were evenly streaked onto TSA agar plates. After growth for 24–48 h, colony numbers were measured (one-way ANOVA; Tukey’s post hoc test, ***p < 0.001, **p < 0.01, *p < 0.05, ns indicates no statistically significant difference).

Compared with the PBS group, the number of colonies in the mixture of *G. parasuis* with mAb 2D1 was significantly reduced (*p* < 0.01), which was not significantly different from the positive control group (Figure 4). In addition, there was no significant difference between groups treated with PBS and mAb ApxIV. The results showed that mAb 2D1 can activate complement, thereby inhibiting the growth of *G. parasuis*.

### Opsonization effect of mAb 2D1 in vitro

**Figure 5:**
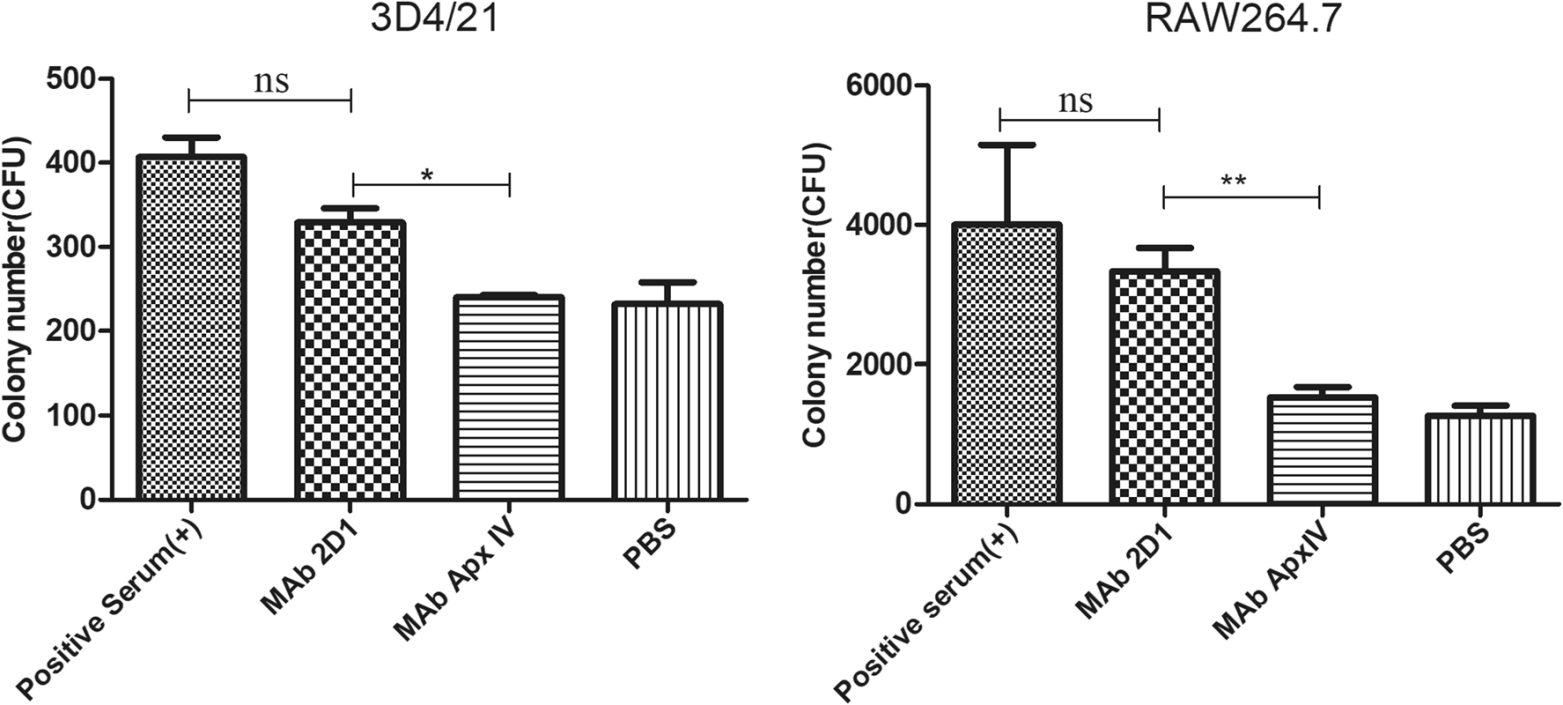
**mAb 2D1 enhances the phagocytosis of 3D4/21 and RAW 264.6 cells to reference serovar 5 of *G. parasuis.*.** After seeding 3D4/21 or RAW264.7 cells into a 12-well plate (approximately 4 × 10^5^ cells/well), *G. parasuis*-5 (incubated with mAb 2D1 for 0.5 h at 37 °C) was added to cells at 200 MOI, and 5% (v/v) guinea pig serum was added to each cell well. After incubation at 37 °C in a 5% CO_2_ atmosphere for 2 h, complete growth medium (including 100 U/mL penicillin G and 0.25 mg/mL gentamicin) was added to each cell well. The cell plate was incubated for another 1 h to kill extracellular bacteria. At the same time, positive serum against *G. parasuis*-5, PBS and mAb ApxIV were used as positive and negative controls. Double-distilled water was used to lyse the cells, and lysates were evenly streaked onto TSA agarose plates. After growth overnight, colony numbers were measured (one-way ANOVA; Tukey’s post hoc test, ***p < 0.001, **p < 0.01, *p < 0.05, ns indicates no statistically significant difference).

Compared with the PBS group, the *G. parasuis* serovar 5 strain incubated with mAb 2D1 was significantly phagocytosed by 3D4/21 cells (*p* < 0.05), which was not significantly different from the positive control group. In addition, there was no significant difference between the groups treated with PBS and the mAb ApxIV (Figure 5). Repeating this experiment with RAW 264.7 cells with stronger phagocytic ability resulted in more significant results (*p* < 0.01). These results showed that mAb 2D1 can enhance the phagocytosis of phagocytes by *G. parasuis*-5 through an opsonizing effect.

### Passive protection of mAb 2D1 in mice

**Figure 6:**
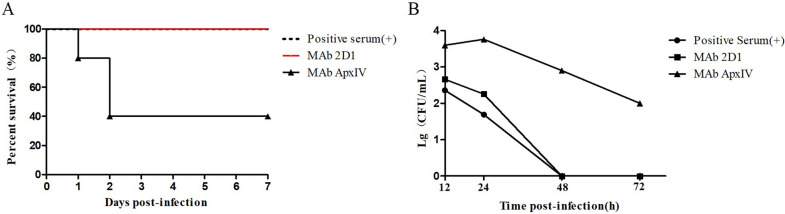
**Passive immunization of mice.** **A** Mice (*n* = 5) received mAb 2D1 intraperitoneally 1 h prior to the challenge. Mice were then challenged with a 2 × LD_50_ dose of 8.42 × 10^9^ CFU of *G. parasuis* by intraperitoneal inoculation. mAb ApxIV was used as a negative control, and positive serum against *G. parasuis*-5 was used as the positive control. Mice were observed for 7 days after the challenge, and the survival rate of mice in each group was compared. **B** Blood bacterial loads of ICR mice after lethal challenge were determined in serial dilutions of 20 µL of blood obtained from the tail vein at 12 h, 24 h, 48 h, and 72 h postinfection in a sterile environment. After being diluted with sterile PBS, the blood was applied to TSA solid medium and incubated at 37 °C for 24 h, and the bacterial load in blood was calculated.

The results showed that all mice passively immunized with mAb 2D1 and positive serum were protected, while the survival rate of mice passively immunized with mAb ApxIV was 40% (Figure 6A). Consistent with the results of the in vivo protection experiment, the mice passively immunized with the mAb ApxIV showed strong levels of bacteremia at 12 h, 24 h, 48 h, and 72 h after the challenge (Figure 6B). At 48 h, the bacteria in the blood of the mAb 2D1 group and positive serum group were completely eliminated. The above results indicate that mAb 2D1 has the ability to clear *G. parasuis* and provides protection against *G. parasuis*-5 infection.

## Discussion

One of the applications of monoclonal antibodies is that they can be utilized to discover predominant immunogens. Since the high purity of immune antigens is a key factor in the preparation of monoclonal antibodies, the traditional preparation method of bacterial-related monoclonal antibodies begins with obtaining the required antigen protein. Because of the structural integrity and immunogenicity of inactivated bacteria, inactivated *G. parasuis*-5 was used to immunize mice and was used as a coated antigen to screen for positive hybridomas by indirect ELISA. mAb 2D1 that reacted with *G. parasuis*-5 and other 14 serovars of *G. parasuis* was screened, so it was used to analyze novel and conserved immunogens of *G. parasuis*.

Using mass spectrometry and Western blot analysis, we identified Fe(3+) ABC transporter substrate-binding protein as the immunogen of mAb 2D1. Iron acquisition is an essential function of most bacterial cells. TbpA and TbpB, which have been reported to have strong immunogenicity, are transferrin receptors; they have specificity for the binding of host transferrin. The tbpA gene encoding TbpA was confirmed to exist in 15 reference serovar strains of *G. parasuis* and clinical isolates of *G. parasuis*. TbpA not only has strong immunogenicity and reactogenicity but can also provide beneficial immune protection against homologous bacteria and different serovar strains in the guinea pig model [16, 17]. Another report showed that the afuA gene, which encodes iron ABC transporter substrate-binding protein, has an 80% protection rate against *G. parasuis* lethal challenge in mice [18]. Fe(3+) ABC transporter substrate-binding protein also belongs to the iron ABC transporter substrate-binding protein clusters, but to our knowledge, the protective efficacy of this protein of *G. parasuis* has not previously been reported.

Humoral immunity plays a major role in the host’s resistance to *G. parasuis* [19], and antibodies are an important part of humoral immunity. IgM, a pentamer with multiple antigen-binding sites, is a high-performance antibody. Its sterilization, lysis, phagocytosis and opsonization are all higher than those of IgG [20]. Because mAb 2D1 is an IgM antibody, its effect on opsonization was assessed. When the complement system is activated by the antigen–antibody complex, the complement CD35 (CR1) receptor on the surface of macrophages binds to IgM, which enhances the phagocytosis of antigens [21]. Passive immunization results showed that the mice containing mAb 2D1 had a stronger ability to eliminate *G. parasuis*-5, suggesting that mAb 2D1 had a protective effect on the attack of *G. parasuis*-5. Data obtained by in vitro evaluation of the complement killing assay and opsonization correlate with the in vivo results; thus, this approach might be valuable to predict protection in vivo.

In summary, using mice immunized with whole bacteria of *G. parasuis* serovar 5, six mAbs were isolated. Of these mAbs, mAb 2D1 against Fe(3+) ABC transporter substrate-binding protein demonstrated opsonization, complement killing effect in vitro and protective activity in vivo, which suggests that this protein could be a target for protective antibodies in mice and may be an ideal and novel immunogen to stimulate immune protection against *G. parasuis* infection.

## Supplementary Information

**Additional file 1.** **Reference serovar strains used in this study.**

**Additional file 2.** **In direct ELISA results of six monoclonal antibodies tested with serovar reference strains of G. parasuis.** Reference serovars 1–15 of *G. parasuis* were used as the coating antigens, six mAbs were used as the primary antibody, and goat anti-mouse IgM/HRP antibody was used as the secondary antibody. The results of indirect ELISA showed that six mAbs can positively react with reference serovars 1–15 of *G. parasuis*, and mAb 2D1 had the strongest reaction with bacteria, so mAb 2D1 was chosen for subsequent experiments.
