# Supplementary material for: Characterization and protective activity of monoclonal antibodies directed against Fe (3+) ABC transporter substrate-binding protein of Glaesserella parasuis
Source: Vet Res. 2021 Jul 5;52:100. doi: 10.1186/s13567-021-00967-1 (PMC8256651; doi:10.1186/s13567-021-00967-1)
Supplement: Supplementary file 1 — Additional file 1. Reference serovar strains used in this study. [file 13567_2021_967_MOESM1_ESM.docx]

**Additional file 1 Reference serovar strains used in this study.**

| 15 *G. parasuis* reference serovar strains | Source |
| --- | --- |
| 1 (NR4) | Japan |
| 2 (SW140) | Japan |
| 3 (SW114) | Japan |
| 4 (SW124) | Japan |
| 5 (Nagasaki) | Japan |
| 6 (131) | Switzerland |
| 7 (174) | Switzerland |
| 8 (C5) | Sweden |
| 9 (D74) | Sweden |
| 10 (H367) | Germany |
| 11 (H465) | Germany |
| 12 (H425) | Germany |
| 13 (IA-84-17975) | USA |
| 14 (IA-84-22113) | USA |
| 15 (SD-84-15995) | USA |
