# Supplementary figures and images for: Characterization and protective activity of monoclonal antibodies directed against Fe (3+) ABC transporter substrate-binding protein of Glaesserella parasuis
Source: Vet Res. 2021 Jul 5;52:100. doi: 10.1186/s13567-021-00967-1 (PMC8256651; doi:10.1186/s13567-021-00967-1)

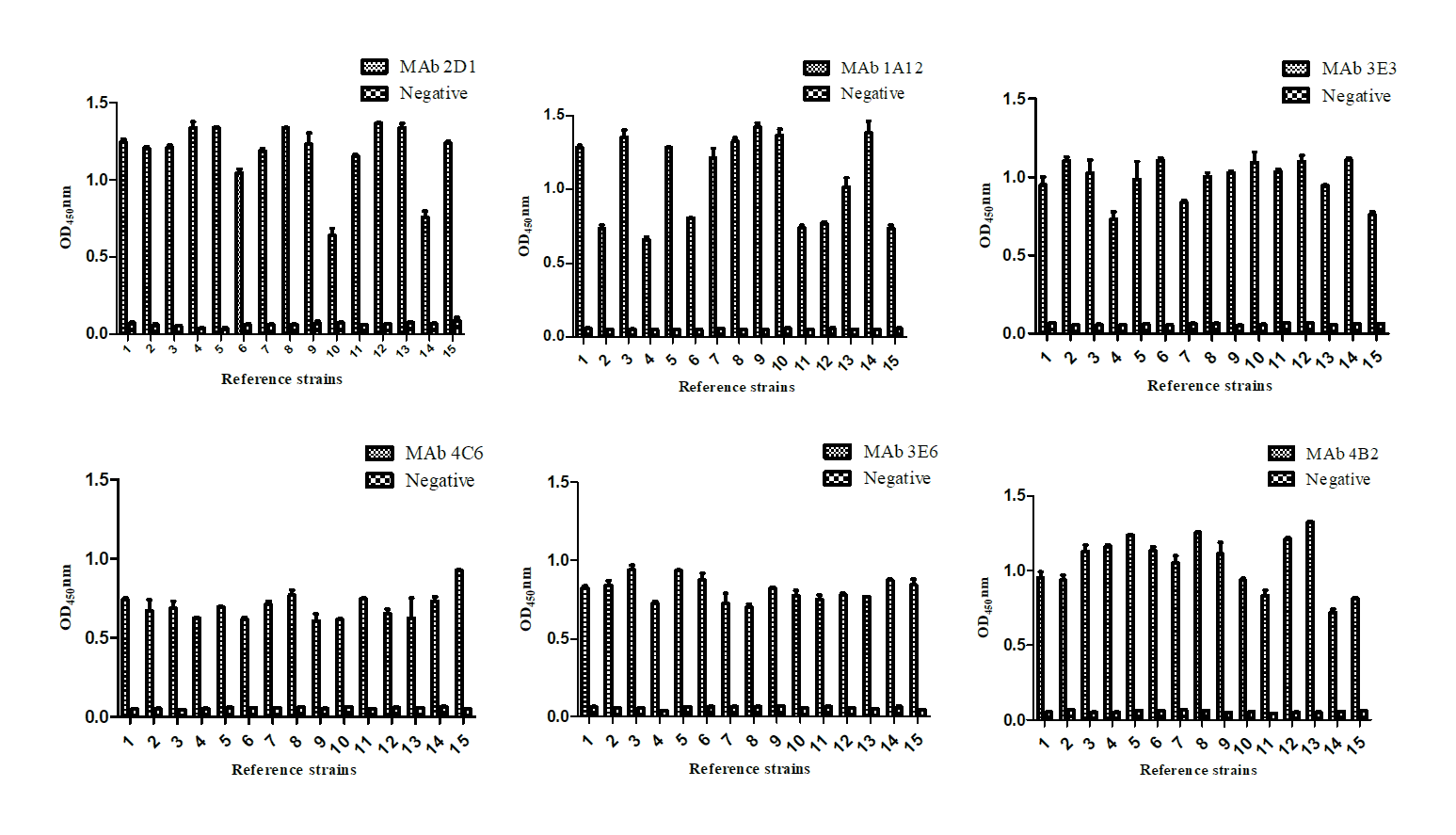

Supplement: Supplementary file 2 — Additional file 2. In direct ELISA results of six monoclonal antibodies tested with serovar reference strains of G. parasuis. Reference serovars 1–15 of G. parasuis were used as the coating antigens, six mAbs were used as the primary antibody, and goat anti-mouse IgM/HRP antibody was used as the secondary antibody. The results of indirect ELISA showed that six mAbs can positively react with reference serovars 1–15 of G. parasuis, and mAb 2D1 had the strongest reaction with bacteria, so mAb 2D1 was chosen for subsequent experiments. [file 13567_2021_967_MOESM2_ESM.tif]
